# Progress in Skin Diseases

**Published:** 1901-05-18

**Authors:** 


### PROGRESS IN SKIN DISEASES.

**Bacteriology of Skin Affections.**—Unna[1] in seventy-four cases of eczema found twenty-three kinds of coccus, two of which, when inoculated into animals, produced typical eczema. One was found in half the cases and the other rarely. Finally both these produced, if inoculated into man, undoubted eczema. On the other hand Veillon[2] thinks that no microbe has been found in the primitive lesions of eczema, but that most of the complications are due to staphylococci and other organisms. Thus Scholtz and Raab found the S. aureus almost always present. Gilchrist[3] comes to a similar conclusion. In impetigo the same writer found streptococci pyogenes in every case and that they could reproduce the disease. They were present also in ecthyma. The pastules of acne are due to a special bacillus and not to common cocci. Pus is also produced by pure cultures of M. ecto and endothrix. Herpes-zoster and erythema multiforme are sterile. Sabourand[4] seems to consider that impetigo contagiosa always contains Fehleisen's streptococcus, but owing to secondary infection staphylococci are added and some of the daughter pustules show only the latter organism.

**Animal Parasites of the Skin.**—G. Pernet[5] has a most interesting paper on the Acaras scabiei and lice. The Acarus is an arachuis and not one of the insecta. In cleanly people and those who handle lime or soda the hands may show no symptoms, though burrows may be plentiful elsewhere, in which the acarus may be found with a lens. The feet in infants are often much affected. For the latter sulphur ointment may be too strong and beta-naphthol, half a drachm to the ounce, may be substituted. Sulphide of potash baths, ℥ij to 30 gallons are useful for adults, but in all cases the skin should be scrubbed with a brush in a hot bath before ointment is applied, and all the clothes, gloves and wrappers must be baked or boiled before being used again. Pediculi do not bite but suck up the blood by means of a haustellum; the impetigo which they occasion is due to a secondary infection of staphylococci where the scalp has been scratched. Acetic acid or vinegar will remove the nits. The hair should be washed or rubbed with 1 in 40 carbolic lotion and afterwards a little white precipitate ointment applied. Crusts should be oiled and removed first. For the P. corposis baking the clothes and stavesacre ointment usually suffices, but weak carbolic baths may be required in obstinate cases. Equal parts of balsam of Peru and simple ointment are recommended for P. pubis. All these affections must be diagnosed from senile and other forms of pruritus and urticaria, dog parasites, and mental hallucinations. Alexander[6] gives three cases of scabies contracted from animals. The disease does not seem to last more than two months, and does not attack the same regions as human scabies. It is usually of a mild type.

**Ringworm.**—Infection from animals is discussed by J. L. Bunch,[7] who shows photos of the cultures of ringworm in eight patients, and of that in the eight animals from whom they were severally infected. His specimens comprise ecto and endothrix varieties and an unknown species. The plate cultures give better means of distinguishing the species than microscopical examination. Thus in growths from the horse the microsporon produces a much denser mass of radiating hyphæ which are individually finer and whiter than those of ecto thrix. Pernet[8] recently showed cultures of the microsporon from a kitten, where the absence of long slender dichotomously branching filaments as seen in large spored forms was evident.

**Eczema.**—Kaposi[9] holds that in a definition of this affection we must include (*a*) an inflammation of the superficial layer of the skin; (*b*) that this can be produced in any person by mechanical and other stimuli; (*c*) that there is itching, (*d*) and the affection goes through a definite course or cycle. Chronic eczema may show a given area affected for a long period, or instead a recurrence in different parts of the skin. Jonathan Hutchinson[10] recommends for most forms of eczema a teaspoonful each of liquor carbonis detergens, and of liquor plumbi subacetatis to a pint of water. This should be kept constantly applied on lint. A stronger preparation may be applied for a few minutes at a time. Leistikow[11] finds that ordinary eczemas of children are best treated by an ointment containing adipis anæ, zinci oxidi, amyli āā 5, hydrarg-oxidi flavi ·25 to ·5, vaselini 10. In the weeping or crusted forms of E. rubrum, he advises the zinc ichthyol salve muslin of Beiersdorf, and in very obstinate cases pyrogallic acid of a strength from $\frac{1}{2}$ to 2 per cent. in the casein ointment. The latter substance contains alkaline casein, water glycerine, and vaseline, and forms a quickly-drying skin easily removed by water. The pyro-gallic acid relieves the itching marvellously, but the urine must be carefully watched. If the skin becomes reddened he returns for a day or two to the ichthyol salve. Naftalein is, according to Hallopeace[12] devoid of irritating properties and applicable in acute cases. In adults it gave him good results and in the seborrhœic eczema of children it surpassed all other remedies. It did great good also in pruritus. Alex. Brownlie[13] speaks highly of the results of ichthyol in both acute and chronic forms internally and externally. Ointments containing from 2 to 10 per cent. and solutions may be employed according to the acuteness of the attack.

**Lichen Planus.**—A most interesting discussion took place at the London Dermatological Society on this subject. Radcliffe Crocker ([14]) describes the lesions as flat elevations bounded by the natural quadrilations of the skin. The epidermis is thickened and raised by exudation beneath it, smooth and shining, at first of a bright red and later on of a violaccous tint. There is a central depression with a horny plug, and sometimes the papules are of irregular shape and convex, and rarely crimson, or ivory-white. The papules itch, and often form lines, occasionally rings, sometimes a long band down a limb (L. striatus), but it is not clear that they follow the track of a nerve. Bullæ may arise and later on pigmentation is usual. The cause of the disease is unknown; though it not unfrequently follows nervous shock or depression. The writer thinks it is quite distinct from L. acuminatus or the so-called Pityriasis r. pilaris, but allied to L. or Parakeratosis variegatus, which, however, is very chronic, attacks even the face, and does not cause itching. The Lichen p. of children is only the remains of miliaria and not a true lichen. Malcom Morris recommends in the terribly severe cases which sometimes occur, biniodide of mercury and emolient baths, or antimony; in chronic ones arsenic with sulphur baths or a rubbing with weak carbolic oil. Pringle notices the frequency with which the mucous membrane of the mouth is attacked, and regards Vidal's L. simplex as merely an irregular form of L. planus. Antipyrin in large doses relieves the itching. Stowers also advocates bromides, followed by quinine and salines. Macleod finds there is an infiltration of the papillary layer, with cells of the connective tissue type such as is seen in the infective granulomata and in lupus erythematosus (Galloway). Ammonol is also recommended in acute cases by Freeman and Unna's zinc paste with perchloride and carbolic externally. Colcott Fox thinks that both the dermis and epidermis undergo inflammatory changes, and in acute cases he employs phenacetin and antipyrin with external remedies similar to the above. Whitfield[15] shows, from sections in the early stage of acute Lichen p., that the epidermal changes are insignificant, but that there is an infiltration of the papillary layer by large cells apparently endothelial in origin. Indeed, in one section such cells could be seen starting from a bud in a capillary vessel. He believes that the irritant causing the disease is imported by the blood stream.

[1] Med. Rec., Nov. 3. [2] Amer. Jour. M. Sc., Feb. [3] Johns Hopkins Rep., Vol. ix. [4] Amer. Jour. Med. Sc., Jan. [5] Clin. Jour., Dec. 26. [6] Amer. Jour. Med. Sc., Feb. [7] Brit. Med. Jour., Feb. 9. [8] Brit. Jour. Derm., Jan., p. 9. [9] Do., Jan., p. 36. [10] Med. Rev. of Rev., Sept. [11] Edin. Med. Jour., Jan. [12] Amer. Jour. Med. Sc., Feb. [13] Lancet, Nov. 24. [14] Brit. Jour. Derm., Dec. [15] Do., Feb.

